# Clinicopathologic features and treatment outcomes of patients with fibrillary glomerulonephritis: A case series

**DOI:** 10.1097/MD.0000000000026022

**Published:** 2021-05-21

**Authors:** Smaragdi Marinaki, Stathis Tsiakas, George Liapis, Chrysanthi Skalioti, Eleni Kapsia, Sophia Lionaki, John Boletis

**Affiliations:** a Clinic of Nephrology and Renal Transplantation, National and Kapodistrian University of Athens Medical School, Laiko Hospital’; b Department of Pathology, Laiko Hospital, Athens, Greece.

**Keywords:** fibrillary glomerulonephritis, histologic features, immunosuppression, outcome, rituximab

## Abstract

Fibrillary glomerulonephritis (FGN) is a diverse glomerular disease with poor renal prognosis. The optimal therapeutic approach remains undetermined, as treatment outcomes vary across different studies.

We retrospectively reviewed the medical data of 10 patients diagnosed with biopsy-proven FGN at our center between 2004 and 2019. Clinical and histological features, as well as therapeutic regimens and treatment response, are reported.

The patients were predominantly men (2.5/1 men-female ratio) with a mean age at diagnosis of 46.5 years (IQR: 41.5-59.5). The median proteinuria and creatinine levels at presentation were 2.55 g/day (IQR: 0.4-8.9) and 1.35 mg/dl (IQR: 0.94–1.88), respectively. Four out of 10 patients presented with nephrotic syndrome, 5 patients with nephritic syndrome and 1 with isolated microscopic hematuria. Light microscopy showed mesangial proliferative (n = 7), membranoproliferative-like (n = 2), and diffuse sclerosing patterns (n = 1). Rituximab was used in 7/10 patients, either as monotherapy (n = 3) or combined with cyclophosphamide and corticosteroids (n = 4). Patients who were treated with immunosuppression had higher median levels of creatinine (1.40 mg/dl) and proteinuria (3.5 g/d) compared to those who received supportive treatment alone (0.94 mg/dl and 0.6 g/d, respectively). After a median follow-up of 30 months (IQR:18–66.5), 4 out of 7 patients (57%) treated with immunosuppression achieved a clinical response, 1 had persistent renal dysfunction and 2 patients progressed to end-stage renal disease.

The present case series extends the existing literature on the clinical features and outcomes of FGN, as well as the use of rituximab-based regimens for the treatment of the disease. Further research is needed to establish the proper management of the disease.

## 1 Introduction

Fibrillary glomerulonephritis (FGN) is an uncommon progressive disease, encountered in 0.5% to 1% of all native kidney biopsies. Lack of discriminating clinical features or serological biomarkers renders kidney biopsy necessary for establishing the diagnosis of FGN.

FGN is characterized by the presence of randomly oriented fibrils in the mesangium and along the glomerular capillary walls.^[1]^ Fibrils are of nonamyloid origin with a diameter of 10 to 30 nm, deprived of a hollow core at a maximum magnification of 30,000 by electron microscopy (EM) examination. Other pathologic findings include a variety of histological patterns by light microscopy (LM) with typically negative Congo-red staining and dominant polyclonal IgG, C3, κ- and λ- light chain glomerular deposits by immunofluorescence.^[2–5]^

Prognosis is generally poor, with approximately half of the patients developing end-stage kidney disease (ESKD) within 5 years from diagnosis.^[4,6]^ Due to the rarity of the disease and the limited number of existing clinical studies, optimal management is not yet determined. Blockade of the renin-angiotensin-aldosterone system is the standard of care treatment, while immunosuppressive therapy has been used with ambiguous outcomes.^[2–4,6,8]^ Clinical data collected over the last decade suggest that the monoclonal anti-CD20 antibody rituximab (RTX) may play a beneficial role in controlling the disease.^[8,11,12]^

In the present case series, we describe the clinicopathological characteristics, therapeutic regimens, and clinical outcome of ten patients with biopsy-proven FGN followed at our institution.

## 2 Methods

We reviewed the medical records of all patients who were diagnosed with FGN between 2004 and 2019. FGN was defined as glomerular deposition of fibrils by EM examination with the following characteristics:

1. random orientation,
2. Congo red-negative stain,
3. diameter of 10 to 30 nm,
4. positive reaction with antisera to immunoglobulins.

LM, immunofluorescence, and examination (EM) was performed in all kidney biopsy specimens. On LM, besides the histologic pattern, the degree of glomerulosclerosis, interstitial fibrosis and tubular atrophy was also assessed.

Following diagnosis, all patients were screened for the presence of monoclonal gammopathy by serum and urine electrophoresis and immunofixation. Immunological work-up also included antinuclear antibodies (ANA), anti-double stranded DNA antibodies (anti-dsDNA), complement levels, antineutrophil cytoplasmic antibodies (ANCA) and cryoglobulin testing. Serologic markers for hepatitis B and C were also performed.

We collected data with respect to the renal function as well as hematological and serological parameters at the time of diagnosis FGN and afterwards. Renal indexes included serum creatinine (sCr) and estimated glomerular filtration rate (eGFR) which was calculated by the CKD-EPI equation, proteinuria quantified by 24 hours urine collections in all subjects, and microscopic urinalysis. Impaired kidney function was defined as a creatinine level above 1.2 mg/dl, and proteinuria as a 24-hour urine protein (Upr) level above 300 mg.

Medical data were recorded from disease diagnosis to the last follow-up visit in the glomerular clinic.

Outcomes of interest included:

1. Complete response was defined as Upr <0.5 g/d and normal sCr,
2. Partial response, as a reduction in proteinuria of ≥ 50% from the maximum value with stable kidney function (<15% decrease in eGFR from baseline),
3. Persistent renal dysfunction, as no response according to previous criteria or progression of kidney disease but not ESKD at the last follow-up visit,
4. ESKD.^[6,8]^

Our study was in compliance with the Declaration of Helsinki and was approved by our Data Protection Officer (No. 120/31-3-2021) and the Ethics Committee of Laiko Hospital. IBM SPSS Statistics 17.0 was used for the statistical analysis of the data.

## 3 Results

A total of 10 Caucasian patients with a median follow-up period of 30 months (IQR: 18–66.5) were included in the study. The median age at diagnosis was 46.5 years (IQR: 41.5–59.5); a male predominance (male to female ratio 2.5:1) was noticed. Patients presented with nephrotic syndrome (n = 4), nephritic syndrome (n = 5) and isolated microscopic hematuria (n = 1). The median Upr excretion of the cohort was 2.55 g/day (IQR: 0.4–8.9), while the median sCr level and eGFR at diagnosis were 1.35 mg/dl (IQR: 0.94–1.88) and 61 ml/min/1.73 m^2^ (IQR: 33–94), respectively. Patients’ demographics and clinical data at presentation are reported in Table 1.

**Table 1:** Demographic data and clinical characteristics at diagnosis.

Cases	Age (y)	Gender	Date	sCr (mg/dl)	eGFR (ml/min/1.73 m^2^)	Upr (g/24h)	Hematuria	HTN	Other medical conditions
1	47	F	2016	1.71	35	8.30	+	+	T2DM + Thyroid disease
2	64	F	2016	3.13	15	1.60	+	+	Thyroid disease + Obesity
3	67	F	2017	0.92	64	0.10	+	+	None
4	42	M	2015	1.00	92	6.70	+	+	Smoking + Obesity
5	46	M	2014	1.40	60	0.22	+	-	Thyroid disease
6	46	M	2015	1.30	65	3.50	+	-	None
7	58	M	2011	2.40	29	0.51	-	+	None
8	47	M	2004	1.70	47	10.80	-	-	None
9	40	M	2018	0.91	105	29.00	+	+	Smoking
10	37	M	2017	0.94	103	0.62	+	-	None

**Figure 1:**
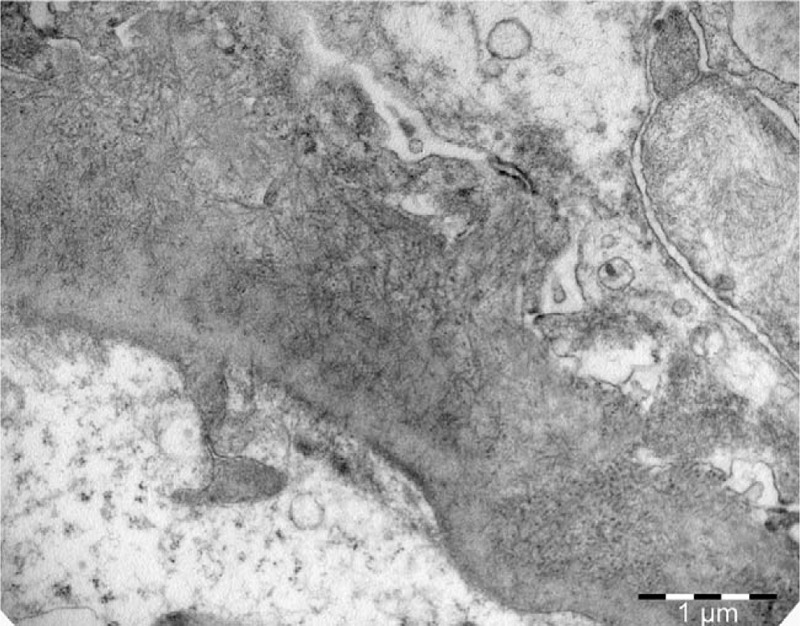
Randomly disposed fibrils into glomerular basement membrane (Uranyl acetate X 18.000).

**Figure 2:**
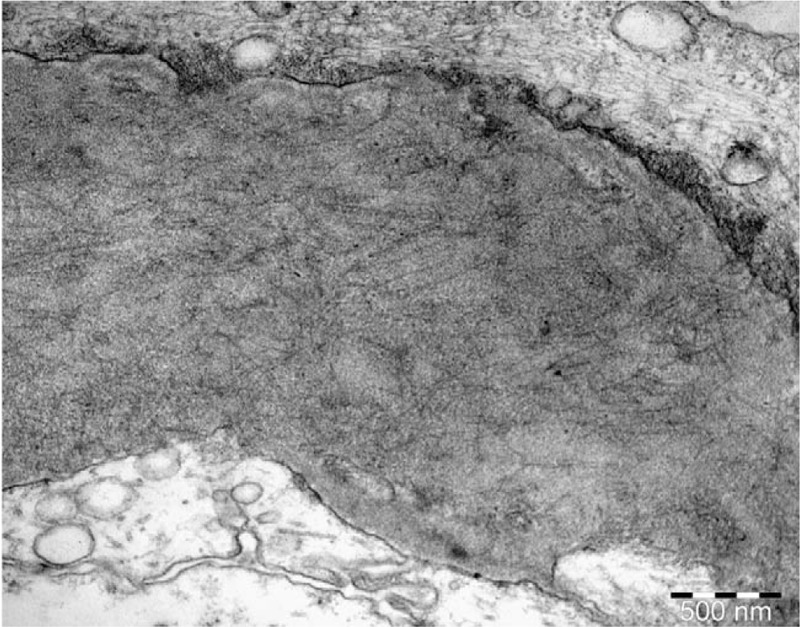
Randomly arranged fibrils infiltrating glomerular basement membrane (Uranyl acetate X 28.000).

**Figure 3:**
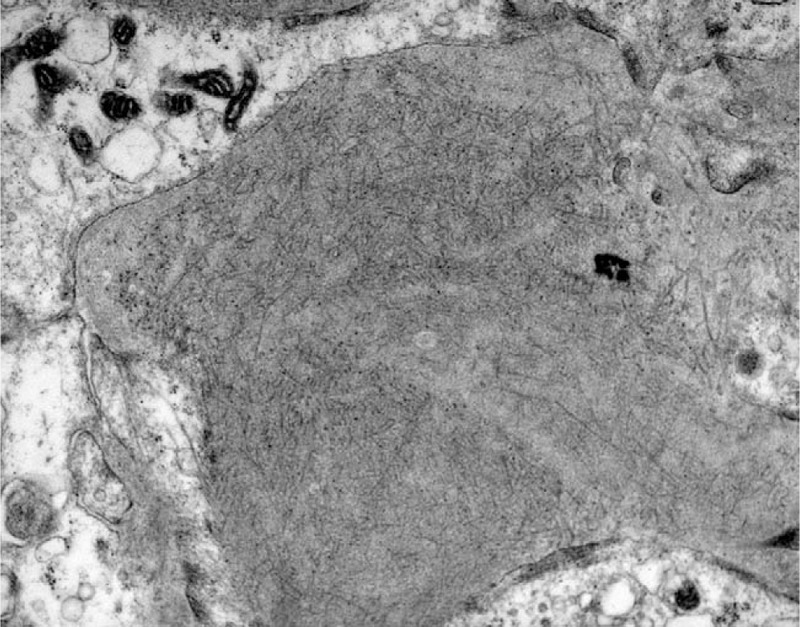
Randomly oriented fibrils in the mesangium (Uranyl acetate X 18.000).

**Figure 4:**
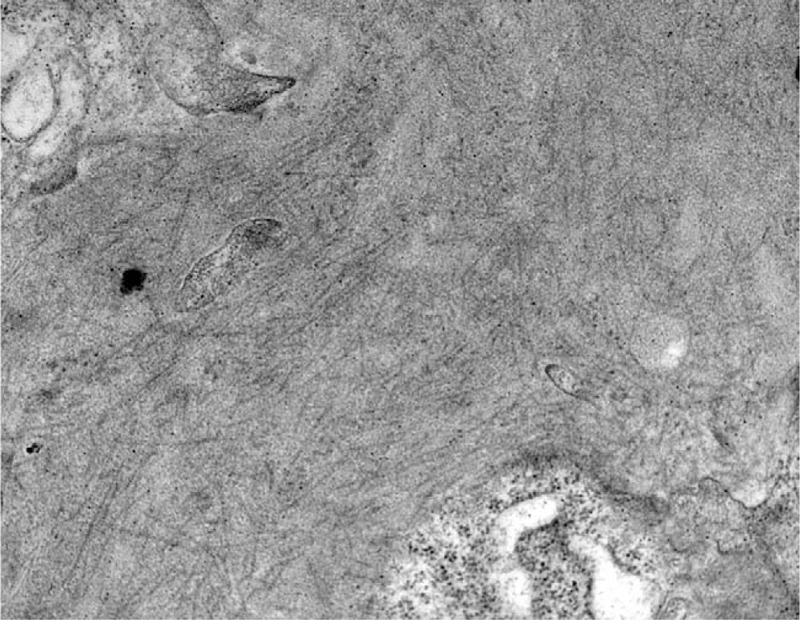
Randomly arranged fibrils in the mesangium (Uranyl acetate X 28.000).

Histological patterns by LM included mesangial proliferative glomerulonephritis (MesGN) (n = 7), membranoproliferative-like/diffuse proliferative glomerulonephritis (MPGN) (n = 2), and diffuse sclerosing glomerulonephritis (n = 1). On immunofluorescence, most patients (n = 9) stained strongly positive for IgG in the mesangium and the glomerular basement membrane with a granular, linear, or smudged pattern and a mean intensity of 2 orders on a scale of 0 to 3. Polyclonal staining for k- and λ-light chains was observed in all kidney biopsy specimens. IgG subtyping was not performed. On EM, except for randomly arranged fibrillar deposits in the mesangium and the glomerular basement membrane (Figs. 1–4), effacement of the podocyte foot processes was also observed in 6 patients (Table 2).

**Table 2:** Histopathological features.

Cases	LM pattern	Global glomerulosclerosis	IF/TA	Crescents	Immunofluorescence
1	MPGN/DPGN	Mild	Mild	2 cellular+ 3 fibrocellular	IgG+, C3+++, C1q++, λ++, κ+
2	DSGN	Severe	Moderate	2 fibrous	IgG+++, C3++, C1q+, κ+, λ+
3	MesGN	Moderate	Mild	−	IgG++, IgA+, C3+, κ+, λ++
4	MesGN	Mild	Mild	−	IgG+++, ΙgΜ++, C3++, κ+, λ++
5	MesGN	Mild	Mild	−	IgG+++, C3+++, κ+, λ++
6	MesGN	N/A	Mild	−	IgG++, C3++, κ+, λ++
7	MPGN/DPGN	Severe	Moderate	−	IgG++, IgA+, IgΜ+, C3++, C1q++, λ+
8	MesGN	Mild	Mild	−	IgG++, C3++, C1q++, κ+
9	MesGN	Mild	None	−	IgG++, IgM++, C3++, κ +, λ++
10	MesGN	Mild	Mild	−	IgG++, IgM++, C3++, C1q++, λ++, κ+

Screening for monoclonal paraproteinemia, chronic viral infections as well as serologic studies for other systemic autoimmune disorders were negative in all cases.

All patients received supportive treatment with renin-angiotensin-aldosterone system (RAAS) inhibitors. Immunosuppressive therapy was applied to 7 patients with high-level proteinuria, impaired renal function, or both. None of the patients had received prior immunosuppression. The immunosuppressive regimen consisted of RTX in all subjects, either as monotherapy (n = 4) or in combination with cyclophosphamide (CYC) and glucocorticosteroids (GC) (n = 3). The latter were used either concomitantly with RTX (n = 3) or later due to inadequate response (n = 1). RTX was used as first line therapy immediately after diagnosis in all patients. RTX was administered in 2 fortnightly doses of 1 g or 4 weekly doses of 375 mg/m^2^, CYC in 6 monthly iv doses of 0.5 to 1 g/m^2^ and GC at an initial dose of 0.5 to 1 g/kg tapered to 5 mg/day by month 3.

### 3.1 Treatment outcomes according to clinical presentation

Four out of 10 patients presented with ***nephrotic syndrome***. All 4 patients were males with a median age of 44 years (IQR: 40.5–46.8). They had a median Upr excretion of 8.75 g/24 hours (IQR: 4.3–24.5) and a median creatinine level of 1.15 mg/dl (IQR: 0.93–1.60). Histological findings on LM included a dominant mesangial proliferative (MesGN) pattern in all patients (No. 4,6,8,9). Diffuse podocyte foot process effacement was also observed in patient 9, who presented with severe proteinuria (29 g/24 hours).

Three out of 4 patients were treated with RTX, CYC and GC (Table 3). Patient 9 achieved partial remission at 22 months and received a second cycle of RTX for maintenance. In patient 4, CYC and CS were introduced 2 years after RTX administration with no impact on his proteinuria as well. His kidney function remained stable until his last follow-up visit at 45 months. Patient 6 progressed to ESKD within 26 months and underwent a living donor kidney transplantation, with no evidence of disease recurrence 9 months post-surgery. Patient 8 was managed conservatively reaching partial remission at 18 months. He developed lung cell carcinoma 12 years after FGN diagnosis, while in remission.

**Table 3:** Treatment and clinical outcomes.

Cases	LM pattern	Follow-up (mo)	Immune suppression^∗^	sCr (mg/dl) initial	sCr (mg/dl) last	Upr (g/24h) initial	Upr (g/24h) last	Outcome
1	MPGN/DPGN	26	RTX + CYC +GC	1.71	1.21	8.30	1.6	PR
2	MesGN	34	RTX	3.13	1.82	1.60	0.28	PR
3	DSGN	18	−	0.92	1.05	0.10	0.16	STABLE
4	MesGN	45	RTX + CYC +GC	1.00	1.20	6.70	8.51	PRD
5	MesGN	58	RTX	1.40	1.08	0.22	0.08	CR
6	MesGN	26	RTX + CYC +GC	1.30	ESKD	3.50	ESKD	ESKD
7	MPGN/DPGN	92	RTX	2.40	ESKD	0.51	ESKD	ESKD
8	MesGN	156	−	1.70	1.08	10.80	2.6	PR
9	MesGN	14	RTX + CYC +GC	0.91	0.89	29.00	10.9	PR
10	MesGN	18	−	0.94	0.95	0.62	1.10	PRD

Five patients presented with ***nephritic syndrome***. There were 3 males and 2 females, with a median age of 47 years (IQR: 41.5–61). They had a median creatinine level of 1.71 mg/dl (IQR: 1.17–2.76) and a median Upr level of 0.62 g/24 hours (IQR: 0.37–4.95).

Patient 1, a 47-year-old female with a history of type 2 diabetes mellitus (DM), presented with rapidly deteriorating kidney function and active urine sediment. Renal histopathology revealed an MPGN-like pattern with fibrinoid necrosis and crescents in more than 30% of the glomeruli while immunofluorescence showed a dominant C3 deposition with a weaker staining for IgG, C1q and light chains. There was no other target organ damage from DM or evidence of diabetic nephropathy on kidney biopsy. She received RTX (4 weekly infusions of 375 mg/m^2^) in combination with CYC (6 monthly doses of 0.75 mg/m^2^) and GC (prednisone 1 mg/kg for the first month with a slow taper, total duration 18 months). She achieved partial clinical remission 3 months after treatment induction. An additional infusion of RTX was given as maintenance therapy when CD 19^+^ B lymphocytes reappeared.

Patients 5 and 10 had mild clinical features at diagnosis and a MesGN pattern on kidney biopsy. Patient 5 had a slightly increased creatinine value of 1.40 mg/dl without significant proteinuria (Upr: 0.22 g/24 hours) and achieved complete response after a cycle of RTX. Patient 10 had normal renal function with a Upr level of 0.62 g/24 hours, which gradually increased to 1.10 g/24 hours, despite treatment with RAAS inhibitors. He refused treatment with RTX. Patient 2 presented with severe renal impairment (Cr: 3.13 mg/dl) in the setting of diffuse sclerosing glomerulonephritis. She received RTX and achieved partial remission within 34 months. Patient 7, who presented with impaired renal function (Cr: 2.12 mg/dl), low-level proteinuria and an MPGN-like pattern on kidney biopsy, developed ESKD 96 months after diagnosis, despite treatment with RTX.

A 67-year-old woman (patient No. 3) presented with ***isolated microscopic hematuria*** of glomerular origin and hypertension which was associated with mild mesangial expansion and hypercellularity on kidney biopsy. As a result, she received conservative treatment with RAAS inhibitors and remained in stable condition.

## 4 Discussion

In this case series, we describe 10 patients with FGN, who were diagnosed in our department during a 15-year period. Presenting features varied from isolated microscopic hematuria and/or low-level proteinuria to nephrotic syndrome, with or without renal impairment, and acute nephritic syndrome, confirming the broad heterogeneity of the disease. In accordance with previous studies, MesGN was the most frequent histological pattern, observed in 70% of the cases. The MesGN pattern correlated with a better-preserved renal function at presentation compared with MPGN pattern. Interestingly, previous reports with repeat kidney biopsies suggested that MPGN pattern may represent an evolutional histological stage in the disease course, thus justifying the worse clinical outcomes.^[8]^ Due to the low number of patients in our study, a relationship between histopathology and renal prognosis could not be established.

To date, no consensus exists regarding the optimal management of FGN. Clinical studies have so far failed to demonstrate a clear benefit of immunosuppression over supportive treatment alone. Patients with low creatinine levels and subnephrotic proteinuria were generally treated with RAAS inhibitors, while immunosuppression was reserved for those with the worse clinical findings at presentation. Additionally, given the retrospective design, the small sample size and the various immunosuppressive drugs that were used in most studies, definitive conclusions could not be drawn.^[2–4,6,13]^ Over the past 2 decades a favorable outcome with the use of RTX has been reported. In 2008, Collins et al described an improvement in proteinuria as well as preservation of kidney function in 3 cases with FGN who received RTX.^[11]^ Further supporting evidence came from a series of 27 patients published in 2013 by Javauge et al, where 5 out of 7 RTX -treated patients achieved a clinical response.^[8]^ Moreover, Hogan et al postulated that the timely initiation of RTX soon after diagnosis may halt disease progression.^[12]^ In 2020, a prospective trial by Erickson et al showed that RTX (2 infusions of 1 g each, 2 weeks apart) at presentation followed by another course 6 months later, led to a decline in proteinuria and stabilization of renal function in 11 patients with biopsy-proven FGN.^[14]^

A RTX -based regimen was used in our patients with impaired renal function and/or high-grade proteinuria. Clinical response was observed in 57% of them, indicating that RTX, as a single agent or as combination therapy, may be beneficial, considering the poor renal prognosis of the disease. In our series, response to treatment did not seem to correlate with sCr levels or the degree of glomerulosclerosis at the diagnostic kidney biopsy, in contrast to previous studies where these factors comprise the main independent predictors of clinical outcome^[8,11,12,15]^. Of note, RTX induced disease remission in case 2, regardless of the elevated creatinine and the high percentage of glomerulosclerosis on kidney biopsy. In contrast, case 6 progressed to ESKD within ∼2 years and case 4 had persistent renal dysfunction, despite their good renal function at treatment initiation.

In our series, 4 patients received CYC and corticosteroids in addition to RTX. The decision for additional immunosuppression was based on the presence of active inflammatory lesions (necrosis or crescents) on kidney biopsy, the clinical severity of the disease or the lack of response to RTX. For instance, case 1 presented with clinical and histological findings of acute glomerulonephritis and achieved clinical remission soon after immunosuppressive therapy induction. In case 4, CYC and corticosteroids were initiated due to non-response to RTX with no additional benefit to his proteinuria.

Kidney transplantation offers a reasonable treatment option for individuals with FGN who reach ESKD. A recent analysis from the ANZDATA registry showed that FGN patients achieved comparable 10-year patient and allograft survival outcomes to kidney transplant recipients with other causes of ESKD. FGN recurred in a single subject (8%), leading to graft loss approximately 9 years post-transplant.^[16]^ Relapse rates were quite higher (36%) in the series by Nasr et al; however only a minority of these patients lost their renal graft as a result of disease recurrence.^[6]^ It should be noted that when FGN is associated with monoclonal gammopathy,^[9]^ at least in the rare cases reported, higher recurrence rates are observed compared to idiopathic FGN.^[17]^ None of our patients had evidence of paraproteinemia or monotypic light-chain deposition by histopathology. We proceeded to a living-donor kidney transplantation in 1 patient who reached ESKD. The patient maintains an excellent graft function with no evidence of disease recurrence 9 months post-transplant.

The pathophysiologic process of FGN has not been fully elucidated. The typical presence of polyclonal IgG glomerular deposition and the reported association with autoimmune diseases, HCV infection or malignancy, indicate that chronic immune system overactivity may contribute to the accumulation of immune complexes and microfibril formation. Whether the presence of other associated medical conditions affects the renal prognosis of FGN is not yet determined. Treatment of the underlying cause though should be pursued in order to achieve the best clinical outcome.^[6,18,19]^ Monoclonal gammopathy, malignancy or HCV infection were not detected in any of our patients, whereas 3 of them had a history of well-controlled autoimmune thyroiditis. Recently, protein DNAJB9, a member of the DNA-J heat-shock family, was identified as a constituent part of the fibrils in FGN.^[20]^ The role of DNAJB9 in the pathogenesis of the disease is not yet clarified; its absence though in amyloid fibrils as well as microtubules, makes it a potential highly specific biomarker for FGN.^[21]^ It is beyond doubt, that the diagnosis of FGN may be missed if EM is not performed. In contrast, the presence of atypical segmental fibrillar deposits could be misleading, since fibrils are encountered in a series of other glomerular disorders, namely diabetic nephropathy, cryoglobulinemic glomerulonephritis and lupus nephritis.^[10]^ The use of mass spectrometry and the discovery of the immunohistochemical biomarker DNAJB9 comprise promising tools for a more accurate diagnosis of FGN.^[20]^ These techniques could be particularly valuable in diagnostic dilemmas.

Our study has several important limitations. Due to its retrospective nature, the small number of included patients and the lack of a comparison group, no firm conclusions can be drawn about the efficacy of each therapeutic regimen on clinical outcomes.

In conclusion, despite its limitations our case series comprised 10 patients with an extensive hematologic and immunologic work-up, detailed histological examination and close long-term follow up, thereby extending the existing literature and further supporting that FGN is an uncommon glomerular disease with a wide variety of clinical and histological features, as well as differing outcomes and treatment response.^[4,6–8]^ RTX, either as monotherapy or in combination with other immunosuppressive agents, appears to be effective in preserving renal function and reducing proteinuria in a significant proportion of patients. Progress has been made in terms of identifying novel tissue and serum biomarkers, that may provide new insights into the pathogenic pathways of the disease.^[22,23]^ Future studies need to determine the factors which are associated with response to RTX or other immunosuppressive agents, the optimal dosage and duration of therapy, and the management of refractory cases with FGN.

## Author contributions

**Conceptualization:** Smaragdi Marinaki.

**Data curation:** George Liapis.

**Investigation:** George Liapis, Eleni Kapsia.

**Methodology:** Sophia Lionaki.

**Supervision:** John Boletis.

**Validation:** Chrysanthi Skalioti.

**Writing – original draft:** Smaragdi Marinaki, Stathis Tsiakas.

**Writing – review & editing:** Smaragdi Marinaki, Sophia Lionaki, John Boletis.
